# Navigating Communication in Nursing Homes During COVID-19: Perspectives From Families, Healthcare Professionals, and Managers in Southern Switzerland—A Qualitative Study

**DOI:** 10.3389/ijph.2024.1606583

**Published:** 2024-10-22

**Authors:** Sheila Bernardi, Maddalena Fiordelli, Sara Rubinelli, Viviana Spagnoli, Roberto Malacrida, Graziano Martignoni

**Affiliations:** ^1^ Medical Humanities, Sasso Corbaro Foundation, Bellinzona, Switzerland; ^2^ Institute of Public Health, Faculty of Biomedical Sciences, University of Italian Switzerland, Lugano, Switzerland; ^3^ Department of Health Science and Medicine, University of Lucerne, Lucerne, Switzerland; ^4^ ADiCASI, Nursing Home Managers’ Association of Italian Switzerland, Camorino, Switzerland

**Keywords:** COVID-19, nursing home, communication, resources, public health measures

## Abstract

**Objectives:**

This study aims to understand the effectiveness and challenges of communication strategies implemented to maintain contact between nursing home (NH) residents and their families during the COVID-19 pandemic, by considering the perspectives of families, healthcare professionals, and NH managers.

**Methods:**

Using a qualitative research design, the study analyzed in-depth semi-structured interviews with key stakeholders (N = 34), including family members, NH staff, and managers.

**Results:**

The study found that communication strategies like video calls, telephone calls, and window visits were generally appreciated and facilitated contact between residents and their families. However, challenges emerged around technical and organizational issues. Both internal and external stakeholders concurred that an increase in technological and human resources was necessary to alleviate these challenges.

**Conclusion:**

The study underscores the importance of innovative and flexible communication strategies to sustain connections between NH residents and their families in crises such as the COVID-19 pandemic. Future readiness calls for increased investment in human and technical resources, and a commitment to understanding and addressing the diverse communication needs of NH residents.

## Introduction

The first wave of the Sars-CoV-2 pandemic hit nursing homes (NHs) tremendously. A significant proportion of NH residents tested positive for the virus, with a large number of COVID-19 related deaths registered in NHs worldwide [1]. Given the scale of the impact, strict public health measures were put in place globally, such as visitor restrictions [2–7]. In Switzerland, where this study takes place, public health restrictions were particularly focused on people 65+, who were most affected by the virus [8, 9] and visits inside the NHs were strictly forbidden, except in situations involving end-of-life care [2, 9]. This lead to reduced social contact for residents and their families [2–7]. Research related to the COVID-19 pandemic, confirmed that NHs closure, with residents’ isolation and loss of social connection with family, friends, and peers, had a negative impact on both physical and mental health in residents [7, 10, 11]. Moreover, functional, cognitive, and nutritional decline was found in nursing home residents after the first wave of COVID-19, in both infected and not infected residents. This support the idea that the decline is not related to the infection itself but rather to the experience of social isolation [12]. Physical distancing and the residents’ physical and psychological state have, in turn, a negative psychological impact on family members and friend’s wellbeing [4, 7]. This is exacerbated in cases where the resident has cognitive impairments [3]. Physical distancing also generates frustration and affects the connection between family members and residents, contributing to a sense of anticipatory grief and ambiguous loss [4, 7, 13].

The negative consequences of such isolation sparked a worldwide effort to develop and implement innovative communication strategies such as an increased use of telephone calls and the introduction of video calls [4, 6, 7]. These initiatives came with several initial challenges like the availability of devices, the lack of appropriate IT infrastructure, low digital literacy of both family members and residents resulting in a constant need of staff presence to ensure the correct use of devices. Window visits were also introduced. The challenge in implementing this measure was the lack of space inside some nursing homes. As a following step, outdoor in-person visits have been permitted. Those require an outdoor location where residents can meet their loved ones while maintaining 2 m of physical distance and wearing a face mask. The use of this PPE often caused communication difficulties between residents and visitors [4, 6, 7, 14, 15]. In the Swiss contest, public policies were more strict with NHs: the nursing home closed in early March 2020 [2] and video and telephone calls, as well as window visits, were introduced by the end of March 2020 [16, 17]. It was only in early June 2020 that visits in presence were introduced [18, 19].

To date, little is known about the impact of these strategies in maintaining and facilitating communication between residents and families during the closure of NHs. The few existing studies suggest that a higher frequency of phone calls between residents and families and email exchange between families and staff, is associated with families experiencing less negative emotions and perceiving better emotional state in NHs residents [20]. Families’ satisfaction with communications strategies increased in relation to the number of possibilities to stay in touch with nursing home residents [4] and in presence visits had a positive impact on residents wellbeing [21]. Some studies suggest also barrier to communication, in using measures such videocall with specific population like patient with sensory and/or cognitive impairment [22, 23].

This study aims to explore the effectiveness of these communication strategies to facilitating and maintaining communication between families and NH residents during the first wave of the pandemic, from the perspectives of families, healthcare professionals, and NH managers.

## Methods

This qualitative study was conducted in the southern and Italian-speaking regions of Switzerland, specifically Canton Ticino and Moesano, from May to August 2020. We worked in collaboration with the Association of Nursing Home Directors of the Italian-speaking part of Switzerland (ADiCASI) to collect data through qualitative semi-structured interviews.

### Study Sample Recruitment

The participant pool included relatives of nursing home residents, healthcare professionals, and nursing home managers. The recruitment took place in close collaboration with one of the authors (VS), training coordinator of ADiCASI. There are 75 nursing homes in southern Switzerland, 72 of which are affiliated with ADiCASI and were approached for participation. We sent letters to all NH managers outlining the study’s purpose and ethical considerations. This letter also extended an invitation to participate to the staff and families of residents. The inclusion and exclusion criteria were defined to ensure representativeness. We specifically sought nursing home managers, doctors, and nurses with either less than 2 years of experience or more than 10 years of experience, as well as family members younger or older than 65 years (with older individuals being more affected by health restrictions). Prospective participants were invited to directly contact the first author (SB) to arrange an interview, independent of the nursing home manager.

### Data Collection and Study Design

Individual semi-structured interviews were conducted in person or via phone between June and August 2020. The interview guide (Supplementary File S1), drafted by one of the authors (SB) and revised by the authors expert in qualitative research (MF and SR), prompted participants to reflect on their experiences during the nursing home closures between March and June 2020. The guide encompassed five sections: ice-breaker questions, attitudes about closure, experiences of closure, communication strategies adopted, and an open comment section. For this study, we concentrated primarily on data from the sections regarding the experiences during the closure and communication strategies adopted.

### Data Analysis

Interviews were transcribed verbatim, and these transcripts were analyzed using an inductive-deductive thematic approach. After a first phase of familiarization with the daty, we reviewed them inductively to identify emergent themes until theoretical saturation was reached. The themes were meticulously identified and organized, leading to the development of a list of codes. These codes were then used deductively to analyze the remaining interviews. Codes were later categorized into broader macro-codes. During the analysis, two researchers (SB and MF) regularly compared notes to ensure the accuracy of the results.

## Results

13 NHs joined the study, 8 NHs managers participated (1 woman and 7 men) together with 20 NHs residents’ family members and 16 NHs healthcare professionals (Table 1). Among them, 5 were doctors (women with more that 10 years of experience), 9 nurses (3 of them were man with more that 10 years of experience), and 2 were animators. Between the 20 NHs residents’ family members, 16 were woman, 4 of them were over 65 years old, and 3 of 4 man were below 65 (see Table 1).

**TABLE 1:** Actual sample of a study on communication in nursing homes during COVID-19, Bellinzona, Switzerland. 2020.

Participants	34
**Family members**	**20**
Female	16
Over 65	4
Under 65	12
Male	4
Over 65	1
Under 65	3
**Manager**	**8**
Female	1
Male	7
**Healthcare professionals**	**16**
Doctor	5
Women more 10 years experience	5
Nurse	9
Women more 10 years experience	4
Women less 2 years experience	2
Men more 10 years experience	3
Animator	2

In bold participants per subgroup.

Of the 13 NHs, 8 were in an urban area and the 5 others in a rural one. The 13 NHs had a different experience of infection from COVID-19 in term of numbers of COVID-19 positives residents and deaths. The interviews duration was minimum 30 and a maximum 50 min.

To compare the internal perspectives of nursing home directors and nurses with the external perspectives of family members on communication measures, the results will be presented in a way that emphasizes both the similarities and differences between these two viewpoints.

### Communication Strategies Adopted During the Pandemic

Both the internal perspective (from directors and nurses) and the external perspective (from family members) identified the same communication strategies between residents and their families. These strategies were implemented in two consecutive phases, aligned with public health policy recommendations and the evolving permissions for visitors to enter nursing homes.

### Initial Phase

In the early phase of public closures of NHs in Canton Ticino (from early March to early June 2020), on-site visits were strictly restricted. As per participant responses, strategies such as video calls, phone calls, exceptional in-person visits, spontaneous long-distance visits, letter and gift exchanges, and window visits were deployed for resident-family communication, as outlined in Table 2.

**TABLE 2:** Direct quotes on strategies of communication between resident and family member, from a study on communication in nursing homes during COVID-19, Bellinzona, Switzerland. 2020.

ID	Direct quotes	Source^a^
S1	And then, I must say that we were quite quick to immediately find a way to get them in touch. We started making video calls with our phones right away, but within just a few days, the director provided these … small tablets, in short, to make video calls	Nurse, female, more 10
S2	Uh well, there was no way to actually enter the house there, and you could only have phone contacts. Then the departments were saying to have the … the tablet that you could use for video calls	Family member, female, under 65
S3	Now the animation team, as well as the physiotherapist and the nurse, are largely involved in these things: video calls, and regular calls too. Then the residents who can use the phone or an app, already do so	Doctor, female, more 10
S4	Always like that, through a person, an assistant, I would call and say “put my sister-in-law on the line” and she would pass her to me, and then she would say “hello, hello!”	Family member, female, over 65
S5	[…] and I granted exceptions to certain people to come in and say goodbye to their loved ones, especially in the final stages of life for those who were dying (sighs)	Nurse, female, more 10
S6	I also knew another lady whose mother was there, and we used to see each other often. Her mother was in the Alzheimer’s ward. This lady … her mother practically passed away gradually and died right during that COVID period, and she and her siblings were allowed to visit their mother one at a time inside the Alzheimer’s section, where she was in bed and couldn't get up anymore	Family member, male, over 65
S7	When they initially imposed the lockdown, it was difficult to keep her indoors. So, they used to tell me to come and take my mom because she was extremely anxious. I would pick her up, not touching her, and maybe walk for an hour on the village streets. Then I’d come back, walking back and forth, or taking routes with no traffic. She would be on the sidewalk, and I would be in the middle of the road	Family member, female, under 65
S8	Actually, for some individuals, the medical director allowed visits in the rooms based on all the cantonal-level criteria, which allowed for a bit of contact. Otherwise, there were some who were just giving up on life. So, there are a whole series of factors to take into account in this sense. And voilà, there are a few individuals who have gone out for birthdays. There are two wives who come, twice a week, and they stay in the room with their husbands because otherwise, it’s difficult for them to be separated	Nurse, female, less 10
S9	Yes, she is on the second floor, and we used to talk from the terrace, you know, they allowed us to do that	Family member, female, under 65
S10	We have a facility that allows most of the rooms, I would say almost all of them, to overlook the garden with a balcony. So, for those who can manage it, having an appointment that doesn't involve any risk, like saying, “I’ll come today at 3 and say hello to my mom from the window,” we’ve come up with this approach. We also have a garden, a closed-off section, which allowed us to be adequately distanced but still visually connected to the family member, although a bit visually distant	Animator, female, more 10
S11	And as I was saying, I make up for it with letters that the ladies read to her. I talk about everything, I talk about my grandchildren. I have 9 grandchildren, so I have many things to share	Family member, female, over 65
S12	Well, every now and then, there would be a drawing from a granddaughter or even gifts. They were in quarantine and then they would come up – a drawing, a dove for Easter, cookies … there was a back and forth of packages, you know. And that was enough	Nurse, female, more 10
S13	We had the opportunity to see our mother again, initially in a designated area for this purpose, with masks and, well, a plexiglass wall that made her visible but prevented direct contact. So, again, we used a mobile phone as a mediator, with the challenges that a mobile phone entails in these situations	Family member, female, over 65
S14	We constructed small sitting areas with plexiglass in the middle, sort of a barrier between the family member and the patient. However, there was visual contact, and we installed a microphone that allowed conversation. So, with prior appointments and maintaining distance between appointments, disinfecting all surfaces between each session, and so on, family members could request a meeting. They could see and hear each other, without physical contact	Nurse, female, more 10
S15	Alternatively, we have a bar with windows on the ground floor that allow the resident, who was inside the closed bar, to communicate with the outside through the glass. At least with a greeting, a momentary connection, even holding a phone in hand, they could see each other	Animator, female, more 10
S16	Yes, the window! Initially, there was the window, twice a week, where we could go at least two times	Family member, female, under 65
S17	And then from there, but quite early, already in May, we transitioned to the garden with the nice weather, right? So, the elderly person is under the pergola, then there’s a barrier 2 m away, and there’s the family member. This happened fairly quickly	Family member, female, under 65
S18	Then, at a later stage, not too long after, now I’m not very good with dates, but well, when it became possible, even physical contact was allowed. Two closed sitting areas with plexiglass were added as additional meeting points. And there are two outdoor points of contact as well, where family members can have physical contact with the resident. Of course, the family member has to wear a gown, have their body temperature checked before arriving, etc. So, in reality, we now have four meeting points – two indoors with plexiglass and two outdoors with physical contact	Nurse, female, more 10

^a^ Source column is organized as follows: role, gender, age (for family member) or years of experience (for healthcare professional).

Video calls emerged as one of the earliest newly-introduced communication means. Initially, caregivers utilized their personal mobile devices for this purpose, which was later replaced by facility-provided tablets (Table 2, Quotes S1 and S2).

Phone calls between residents and family members were another commonly used form of communication from the onset of the NHs' closure. This mode of communication, typically used by residents with personal mobile phones before the pandemic, was extended to all residents, facilitated often by caregivers (e.g., physiotherapist, animator) whose regular duties were disrupted due to preventive measures (Table 2, Quotes S3 and S4).

During the closure NHs allowed exceptional in-person visits for critically ill residents nearing end-of-life (Table 2, Quotes S5 and S6), and for those who were significantly struggling with isolation (Table 2, Quotes S7 and S8).

In an attempt to maintain contact, family members also arranged spontaneous long-distance visits, leveraging NH architectural features and outdoor areas such as balconies and gardens (Table 2, Quotes S9 and S10).

Further, caregivers and family members kept contact through the exchange of letters and gifts (Table 2, Quote S11). Family members mainly mentioned exchanges of letters and cards with residents, while caregivers also mentioned the exchange of gifts and food (Table 2, Quote S12).

NHs established designated meeting stations within the facilities where residents and family members were separated by a plexiglass wall, often with a phone present to facilitate conversation (Table 2, Quotes S13 and S14).

Alternatively, meetings could be held with glass windows separating residents inside the building from family members outside, with telephones supporting the conversation in these instances as well (Table 2, Quotes S15 and S16).

### Subsequent Phase

After the initial phase, the government allowed the gradual resumption of in-person visits to nursing homes starting on June 8, 2020 [18]. In this updated scenario, meetings with residents outside the nursing homes were permitted, with mandatory social distancing in place. Alternatively, closer proximity was allowed under strict hygiene protocols, such as wearing masks and gowns. This adjustment even made physical contact permissible, provided that strict hygiene measures were followed (Table 2, Quotes S17 and S18).

### Assessment of Communication Measures

Family members, directors, and nurses generally found the aforementioned communication strategies to be beneficial for maintaining dialogue between residents and their families, despite encountering some challenges (refer to Tables 3–5).

**TABLE 3:** Direct quotes on facilitators to communication between resident and family member, from a study on communication in nursing homes during COVID-19, Bellinzona, Switzerland. 2020

ID	Direct quotes	Source^a^
F1	We installed many glass panels, and this allowed us to facilitate communication through the glass. So, family members with phones on one side and residents with phones on the other could have video calls	Doctor, female, more 10
F2	I have to say that a couple of times I managed to make a video call because, um, with the help of the staff inside the elderly care home, they suggested I do a video call (pause). So, this was truly wonderful and positive because I was able to at least see her, and (pause) it helps, you know	Family member, female, under 65
F3	Among the various options that emerged again for meeting, I believe the one that made the most sense was the possibility of physical contact, even just placing a hand on their shoulder or giving them a hug. For someone like my father (pause), it means much more. There’s much more communication happening physically than verbally, as he is both deaf and certain concepts don't (pause) immediately get through anymore	Family member, male, under 65
F4	Then we immediately started with video calls, which clearly lowered everyone’s anxiety levels because they could see each other and assess from the visual cues whether the person was well, if there were any changes, if they looked pale, happy, sad, or whatever. It wasn't solely based on mutual trust but also had a tangible proof of how their resident was doing	Manager, female
F5	I reiterate that I was happy that I could see her, if she was doing well, if she was dressed nicely, even that. Not that they don't treat them well, but sometimes nobody goes and (pause) that thought crosses my mind. When I go there and see that something is off, I always tell them	Family member, female, under 65
F6	Yes, they were well received by both the family members and the residents (pause). Of course, as I mentioned, those with cognitive deficits struggle to understand “why can't I touch,” but they could still communicate, and that definitely reassured them that “they haven’t forgotten me because they still come to see me,” even if they can no longer have physical contact. So, they were positively received	Nurse, male, more 10
F7	I call her regularly twice a week, and (pause) for her, it’s more rewarding because if she wants to say something, she tells me more calmly (…) yes, because at least those 5 minutes of distraction, she talks afterward, if she’s in the mood for it, in 30 seconds flat (laughs). It depends on her mood (laughs), but afterward, I might hear her smile, and then she always thanks me (sighs). It’s a bit difficult, you know	Family member, female, under 65
F8	However, the directive allowed this in specific situations, always with the involvement of the medical director, as it always happened. But, once it was over, the family members were usually present shortly after the passing, so they were always there at the moment of departure. But the first thing they told me as they left was “Thank you,” all of them, not just one but all of them, “Thank you.” That was their initial reaction. Being able to share that last 5, 10, 30 min, an hour of their loved one’s life – “Thank you.” This is something that’s difficult to negotiate, so to speak	Manager, male
F9	[Interviewer]: Yes. Do you know if this measure satisfied this lady? [Respondent]: Absolutely. Very much so. Especially because there were several siblings, so they could practically take turns visiting her every day, you know. And everyone was able to be close to their mom during this time of … the end of life, you could say. It’s worth mentioning that … um … this lady practically didn't recognize any of her children anymore. So, in the end, it was the same for her whoever came, but not for them. This is important. Because she’s their mom.”	Family member, male, over 65

^a^ Source column is organized as follows: role, gender, age (for family member) or years of experience (for healthcare professional).

**TABLE 4:** Direct quotes on obstacles to communication between resident and family member, from a study on communication in nursing homes during COVID-19, Bellinzona, Switzerland. 2020.

ID	Direct quotes	Source^a^
B1	They had suggested these video calls, but every time I called for a video call, it was never available. So, I would say, “Give me the phone.” What am I supposed to do? I’m not going to call countless times just to get … by now, if there’s only one tablet, and everyone needs it, it was a bit difficult to even … communicate, I think	Family member, female, under 65
B2	For us, it was simply a matter of understanding, as we didn't have Wi-Fi. So, we had to figure out and buy SIM cards for phones; we didn't have anything already installed. In that case, since I’m not very knowledgeable about this, we had to involve our local IT specialist who guided us on what to do. Within six or 7 days, we resolved the issue, and then we could go get the devices, everything that was needed physically. Even there, it needed to be a quick response – “Yes, in a month” wouldn't have been suitable, you know	Manager, male
B3	Visits were very frequent; there were family members coming every day. So, it’s clear that even video calls couldn’t happen every day because each facility had around 70 residents, so we also had to maintain continuity in care. Video calls were arranged, but not on a daily basis with all family members. The big challenge was that family members had to adhere to certain time frames or plan for the next week for the next video call	Manager, male
B4	You can’t go to dinner anymore, I’m fine with that, but certain things, certain meetings should be arranged individually and not limited to these 45 min which are just too tight. Because 45 min, you know, I mean, while working 100%, working in Bellinzona, it’s not like I can just go up there. I can manage a couple of days a week, but my sister-in-law is always there, there’s always someone. But I mean, I’m not at home all the time, so I can’t go at any moment, and I have to adhere to their … their schedules. Do you understand?	Family member, female, under 65
B5	However, these video calls were set up, and they were generally well-received. There, the challenge is a bit for those who don't hear well, those who don’t see well, or those who are a bit confused and don’t understand what it is. It could possibly complicate the situation, you know	Doctor, female, more 10
B6	Given this, as I mentioned earlier, it’s a bit difficult to communicate with a person who has Alzheimer’s through a video call, you know	Family member, female, over 65
B7	With my mom, it’s a bit more problematic in terms of communication because she doesn’t hear well even on a regular phone call; she struggles to understand. And there was a small issue because communication with her didn't happen for a certain period	Family member, male, over 65
B8	Well, I’ve suffered because, um, I regularly talked to my mom, but evidently, um (pause), due to having quite a severe condition – she has a serious neurodegenerative disease – it’s difficult on the phone (pause). It’s really, really tough, so, um (pause), how can I put it, yes, of course, maybe she enjoyed hearing my voice, but (pause) she can’t respond. It’s not like I’m having a normal interaction with her; it’s very different	Family member, female, under 65
B9	You see, there are small gestures. Maybe [the family member] tends to lower the mask to make themselves recognizable because even there, seeing the eyes is fine, but facial expressions are not the same	Nurse, male, more 10
B10	Now they don't wear gloves anymore, but until last week, it was a cap, mask, gloves, and gown … when they came in with an older adult, the elderly person would get scared. (…). The first time it happened, I was dressed like that, and … and she didn't recognize me. She can't recognize, and let alone … so she got scared, tensed up, became nervous, and I had to call the staff to take her to her room	Family member, male, under 65
B11	For me, the only way to connect with my mom is through touch, caressing her. But now, even that is restricted; you have to be careful, wear a mask. Then she always asks me why I’m wearing the mask, she says, “What’s on your face?” Most of the time, she ends up pulling it off. She doesn’t understand, and this is also distressing – not being able to kiss her, not being able to touch her. That’s the only way I have to communicate with my mom, and I can't do it. Okay, fine, for me, just seeing her is enough, what else can I do? But, well (sighs)	Family member, female, under 65
B12	The duration of 45 min is indeed quite long in some situations, and even here, not only have the bookings for visits decreased, but also the length of each individual visit. Now we have an average duration that doesn't exceed 20 min because topics are exhausted fairly quickly. On the other hand, we also have cases of residents who are finding it increasingly difficult to sustain a conversation, and this has led to a gradual decrease in both the number and duration of visits	Manager, male
B13	We started to gradually open up in this sense as well. For some, it worked very well, but for others, not so much because there’s not much familiarity with using these tools. So, what do I look at here? Oh, it’s me, yes, they see themselves reflected	Animator, female, more 10
B14	Well, it was difficult for my aunt to have a phone placed in front of her and (pause) try to get her to talk. I could see she was looking everywhere except at the phone	Family member, female, under 65
B15	But even with the video call, at first, she might recognize you, but then … and then, yes, it’s a video call, but there was always someone else present. So, she would look at the other person and say, “Am I doing it right?” It’s a bit like that, the communication was always tied, well, I can't say many things to my mom, like “How are you?” and those things, and that’s it. Because you can’t have a longer conversation with her. She doesn’t keep up with the conversation on those matters. But let’s say there was no privacy. And it’s still the same with these conversations. Because you’re downstairs, and there’s a separation that divides you, and you hear what others are saying, and they hear what you’re saying. I don’t share any secrets, for heaven’s sake. But the other time, there was a lady downstairs who couldn't hear well and kept shouting all the names of the relatives, and I had to speak into my mom’s ear, otherwise my mom wouldn't understand (laughs)	Family member, female, under 65
B16	I had brought her a flower, they told me it was allowed to bring one, but she didn't receive the plant. I had also written a little note with a small thought from my brother, and she never received it … it’s these things that are missing	Family member, female, under 65
B17	Well, now, at the moment, um … let’s say (sighs) … my thought is for cases like my mom’s, that is, with dementia, with real difficulties … because my mom resists going to the meeting points. Because she has her … outside her room and her floor, she struggles to … loses her bearings, and so she opposes it, even though she sees us in the garden, she sees us in these meeting rooms, but she’s angry there, a bit disoriented	Family member, female, under 65

^a^ Source column is organized as follows: role, gender (m male, f female), age (for family member) or years of experience (for healthcare professional).

**TABLE 5:** Assesment of facilitator and obstacles to communication by internal and external viewpoint, from a study on communication in nursing homes during COVID-19, Bellinzona, Switzerland. 2020.

Internal viewpoint	External viewpoint
**Facilitator to communication**	
Maintain contact between family member and resident	
Reassure the family member	
Reassure the resident	
Enable end-of-life support	
**Obstacles to comunication**	
Difficulties in activating the communication measures	
Limited meeting in time and frequency	
Barrier to non-verbal communication	
Difficult usability for elder	
	Privacy issues
	Denied exchange of gifts and food
	Meeting spaces not conducive to the disorientation of the elder

### Communication Enhancements (or Facilitators)

From an internal viewpoint, strategies such as video calls and indoor visits, facilitated by plexiglass or glass windows, enabled continuous contact between residents and their families despite the closure of NHs. Both managers and caregivers perceived that these communication methods were well-received by family members (Table 3, Quotes F1 and F2).

Family members greatly valued the outdoor visitation areas, which allowed them to personally meet with the residents, touch them, and engage in activities other than conversation. This was particularly important for family members of residents suffering from cognitive decline or hearing impairment (Table 3, Quote F3).

There was a consensus between the internal and external perspectives that the use of video calls provided reassurance to family members. They were able to see their loved ones, observe their physical and emotional wellbeing, and assess the quality of care being provided to the elderly individuals (Table 3, Quotes F4 and F5).

Participants also pointed out that telephone calls and video calls helped reassuring the residents that they were not forgotten by their family members, despite the lack of visits (Table 3, Quotes F6 and F7).

Family members greatly appreciated the opportunity for exceptional visits. These instances allowed them to accompany the resident through their end-of-life stages (Table 3, Quotes F8 and F9).

### Obstacles to Communication

Both managers and family members expressed concerns about the technical and organizational challenges associated with implementing communication measures. Issues included the limited availability of devices within the facility, which reduced the frequency of meetings, and the initial absence of a Wi-Fi network, which took time to activate (Table 4, Quotes B1 and B2).

Both external and internal perspectives highlighted an issue with the limitations on the frequency and duration of video calls, indoor visits with plexiglass or glass windows, and outdoor in-person visits. Video calling wasn't feasible every day and was time-restricted, as were the indoor visits with plexiglass.

Some NHs only allowed outdoor visits on weekdays for 30–45 min with only 1 or 2 persons at a time. This proved challenging for family members who worked during the week and for those who visited residents frequently.

All participants identified time restrictions as a source of dissatisfaction for family members (Table 4, Quotes B3 and B4).

While video calls provided some reassurance to family members (as discussed below), maintaining a conversation with the resident through a device was often difficult, especially when the resident had cognitive decline or sensory impairment (Table 4, Quotes B5 and B6).

As noted by the external perspective, telephone communication also proved challenging for residents with hearing loss and cognitive impairment (Table 4, Quotes B7 and B8).

Indoor visits with plexiglass and phones, and outdoor visits with enhanced hygiene measures (distance, mask, gown), impeded nonverbal communication, especially for residents with cognitive decline (who found it challenging to recognize family members) or hearing loss (who found hearing and lip-reading difficult) (Table 4, Quotes B9 and B10).

Participants mentioned struggles in maintaining dialogue with residents, particularly those with cognitive decline, during indoor and outdoor visits, and expressed difficulties in communication due to the lack of touch and physical proximity or engagement in non-verbal activities (Table 4, Quotes B11 and B12).

For residents unaccustomed to the use of such technologies, handling tablets and phones for video calls proved challenging, resulting in less spontaneous interactions between them and their families (Table 4, Quotes B13 and B14).

Family members pointed out three issues that were not raised by caregivers and managers. Firstly, they mentioned issues of privacy stemming from the presence of an operator during video calls, giving the impression that their conversations with the residents weren’t private. The use of shared spaces for indoor visits with plexiglass also led to communication difficulties due to noise from other visitors and a lack of intimacy (Table 4, Quote B15).

Secondly, family members who were accustomed to exchanging food and gifts with residents found that these exchanges were prohibited due to mandatory quarantine for items entering the facility, making the exchange of fresh food and flowers challenging (Table 4, Quote B16).

Lastly, from the external perspective, it was noted that older adults found it difficult to meet their family members in unfamiliar settings, such as outdoor visitation areas or indoor areas with plexiglass. These environments added to their disorientation (Table 4, Quote B17).

### Lessons Learned

The preceding section highlighted areas for improvement. Consequently, participants were asked to propose ways that NH could better address the challenges in maintaining communication between family members and residents. While some suggestions were specific to the lockdown and COVID-19 measures and not universally agreed upon, there was a consensus on the need for NH to enhance their resources, both in terms of technology and staffing. Participants suggested the inclusion of a dedicated person, such as a volunteer (civilian or military) or an activity coordinator, who could focus solely on facilitating communication, thereby reducing the burden on caregivers (Table 6, Quotes L1 and L2).

**TABLE 6:** Direct quotes on lessons learned, from a study on communication in nursing homes during COVID-19, Bellinzona, Switzerland. 2020.

ID	Direct quotes	Source^a^
L1	Perhaps it wouldn't have been a bad idea to have someone within the team exclusively handling video calls, you know. (…) So that you, as the primary caregiver, wouldn't be taken away from the unit. That would have been a good thing. Like now, we have visits in the garden, and what do they do? They call us, we have to bring the elderly person down, then they call us again, and we have to bring them up, and this disrupts our routine. We would need someone specifically assigned to this task. I don't know, a rotating activity coordinator, a rotating volunteer who takes care of this, so that you can always focus on your unit work. That would have been interesting	Nurse, female, more 10
L2	Exactly, maybe getting someone from the army or civil defense involved, this person could provide support for these visit matters. Even volunteers and such. You could make them available and see if such an arrangement could be implemented, so as not to burden the staff	Family member, female, under 65
L3	Well … it could have been done, it could … it could still be done, perhaps further increasing the use of video calls. But on a technical level, I don’t know how to do it either, you know. Creating more autonomy for the residents to give them the opportunity to talk, but these are things that perhaps should have been considered earlier. Thinking about a little device that each resident could have in their room to contact their family and *vice versa* at any time, instead of depending on these scheduled appointments, on these things, you know	Nurse, male, more 10
L4	Exactly, there’s room for improvement in things. Even in communication. Maybe instead of having just one tablet, have one tablet for each floor, or two tablets for each floor	Family member, female, under 65

^a^ Source column is organized as follows: role, gender (m male, f female), age (for family member) or years of experience (for healthcare professional).

The need for improved technology for remote communication was also underscored, such as the provision of additional tablets. This could enable more frequent interactions between family members and residents (Table 6, Quotes L3 and L4).

## Discussion

This study aimed to examine the perceived effectiveness of communication strategies used to maintain interactions between families and nursing home (NH) residents during the first wave of the COVID-19 pandemic. We took into account the perspectives of families, healthcare professionals, and NH managers. Our findings indicated that the unique circumstances necessitated flexible implementation of both conventional and novel communication methods. There was an uptick in phone calls and exchanges of letters and gifts, while video calls, window visits, and outdoor meetings with enhanced hygiene measures were newly introduced. NHs ensured special visitations for residents nearing end-of-life, and families took the initiative to arrange unplanned, long-distance visits. All participants agreed that, despite some challenges, these measures helped maintain contact, provide reassurance to all parties, and facilitated end-of-life support.

Firstly, the use of telephone calls, video calls, and increased hygiene measures (such as physical distancing and facial masks) proved a communication barrier for those suffering from sensory and/or cognitive decline—a challenge well-documented in existing literature [22, 24]. Consequently, electronic devices like tablets were not independently usable by older adults. This required the presence of nursing home staff, which affected residents’ and families’ privacy and increased the caregivers’ workload.

Secondly, NH staff faced difficulties in ensuring regular meetings, with time and frequency limitations posing an issue. Lastly, technical and structural resources were problematic. The initial absence of Wi-Fi and electronic devices delayed video call implementation, while meeting spaces inside NHs failed to provide necessary privacy and were unsuitable for disoriented older adults.

Despite acknowledging the benefits and challenges of the measures undertaken, family members were more critical, highlighting further issues. Prior studies suggest an alignment between family members and NH staff on assessing residents' needs, but they also observed that nurses often perceive residents’ problems as less problematic than family members do [25].

Our findings highlight the crucial role that both NH staff and family members played in implementing various communication methods, as well as the adaptability of NH workers. While existing literature emphasizes the central role of NH managers and staff, the contributions of family members in suggesting new communication modalities are still largely unexplored. Often, NHs purchased tablets to facilitate video calls [26]. Care and activity teams collaborated to support communication between families and residents, with NH care staff assisting with device usage and activity teams aiding in writing letters [26, 27].

Additionally, NH managers and staff regulated exceptional visits [28] and regularly updated families on residents' wellbeing [29]. This added to NH staff’s workload [21, 28, 30], already burdened with infection control and emotional stress due to residents' loneliness, illness, and death [10, 31]. Our results suggest that re-evaluating technical, human, and structural resources in nursing homes is essential for effectively managing future crises, reinforcing findings from previous studies [6].

Communication is inherently linked to quality of life (QoL) not only for family members [3] but also for NH staff [31] and, most importantly, for the residents themselves [32]. Enhancing technical and human resources can maintain relationships outside NHs [6] and facilitate staff-resident interactions and physical activities [33]. Moreover, architectural considerations can enhance both internal and external communication, resident privacy, and social activities, all contributing to QoL [34]. The scarcity of human resources is a known ethical challenge in NHs [35], relating to principles of non-maleficence and distributive justice [36], and the concept of dignity [37]. Thus, our findings, albeit limited to the COVID-19 period, underscore an existing need for NHs to increase human, structural, and technical resources in order to ensure meaningful change in communication within nursing homes. We argue the need to place communication at the center of care practices to create an ethical care environment [36], ensuring that all parties are actively involved and held accountable [38, 39].

### Limitations

Our research faces four primary constraints. The first is linked to the study design, which doesn’t encompass a longitudinal view or quantitative data. A more in-depth exploration of how communication measures developed during the pandemic, along with potential additional difficulties or solutions, could be insightful. Incorporating quantitative data could have offered a more comprehensive understanding of the effectiveness of communication strategies. Yet, this investigation aimed to initially inform regional public health policies about challenges faced during the initial implementation of communication measures (press conference in December 2020), prioritizing an examination of the prevailing circumstances.

Secondly, our study is geographically limited to the southern, Italian-speaking region of Switzerland, which may restrict the applicability of our findings to other cultural contexts. Nevertheless, research conducted in various countries corroborates a similar progression of events, communication measures enacted, and associated challenges. Given that the area we examined was severely affected by the pandemic, we argue that our findings could be relevant in other contexts.

A third limitation may be the reliance on nursing home managers for participant recruitment. However, participants were encouraged to contact one of the authors (SB) directly, which helped ensure their anonymity.

The last limitation is the omission of resident’s viewpoints on the implemented communication measures. As of now, however, no study has compared internal and external perspectives on residents’ relational wellbeing and communication during the COVID-19 pandemic.

### Conclusion

This study illuminates the critical role that communication plays in the care of nursing home residents, particularly under the stressors imposed by the COVID-19 pandemic. Our findings underscore the benefits of various communication measures in maintaining connections between residents and their families while also outlining the difficulties experienced in their implementation. Significantly, these findings highlight the necessity for nursing homes to augment both human and technological resources to facilitate smoother communication. Despite the limitations inherent in its geographic focus and study design, the study provides valuable insights that extend beyond the specific context of the COVID-19 pandemic. Ultimately, it underscores the fundamental need for nurturing human connections in care environments and the critical role of adaptability in overcoming unprecedented challenges. It is a call to action for ongoing investment and innovation in the domain of nursing home communication, ensuring a high quality of life for residents, their families, and the staff who care for them.

## References

[1] World Health Organization. Preventing and Managing COVID-19 across Long-Term Care Services: Policy Brief, 24 July 2020 (2020). Available from: https://www.who.int/publications-detail-redirect/WHO-2019-nCoV-Policy_Brief-Long-term_Care-2020.1 (Accessed August 22, 2023).

[2] Casa Anziani Girasole. Direttive del Medico Cantonale Sull’accesso Alle Case Per Anziani (2020). Available from: https://www.massagno.ch/Direttive-del-Medico-cantonale-sull-accesso-alle-Case-per-Anziani-9bdf9d00 (Accessed July 28, 2021).

[3] O’Caoimh R O’Donovan MR Monahan MP Dalton O’Connor C Buckley C Kilty C Psychosocial Impact of COVID-19 Nursing Home Restrictions on Visitors of Residents With Cognitive Impairment: A Cross-Sectional Study as Part of the Engaging Remotely in Care (ERiC) Project. Front Psychiatry (2020) 11:585373. 10.3389/fpsyt.2020.585373 33192731 PMC7649131

[4] Wammes JD Kolk MSD van den Besselaar MJH MacNeil-Vroomen PhD JL Buurman- van Es Rn BM van Rijn PDM. Evaluating Perspectives of Relatives of Nursing Home Residents on the Nursing Home Visiting Restrictions During the COVID-19 Crisis: A Dutch Cross-Sectional Survey Study. J Am Med Dir Assoc (2020) 21(12):1746–50.e3. 10.1016/j.jamda.2020.09.031 33148480 PMC7524682

[5] Trabucchi M De Leo D. Nursing Homes or Besieged Castles: COVID-19 in Northern Italy. Lancet Psychiatry (2020) 7(5):387–8. 10.1016/S2215-0366(20)30149-8 32353267 PMC7185948

[6] Ickert C Rozak H Masek J Eigner K Schaefer S. Maintaining Resident Social Connections during COVID-19: Considerations for Long-Term Care. Gerontol Geriatr Med (2020) 6:2333721420962669–5. 10.1177/2333721420962669 33110931 PMC7557236

[7] Paananen J Rannikko J Harju M Pirhonen J. The Impact of Covid-19-Related Distancing on the Well-Being of Nursing Home Residents and Their Family Members: A Qualitative Study. Int J Nurs Stud Adv (2021) 3(100031):100031. 10.1016/j.ijnsa.2021.100031 34095858 PMC8166157

[8] Ufficio federale della sanità pubblica (UFSP). COVID-⁠19 Svizzera | Coronavirus | Dashboard (2020). Available from: https://www.covid19.admin.ch/it/epidemiologic/death (Accessed July 14, 2022).

[9] Radiotelevisione Svizzera Italiana (RSI). Ticino: 834 Contagi, 22 Decessi (2020). Available from: https://www.rsi.ch/news/ticino-e-grigioni-e-insubria/Ticino-834-contagi-22-decessi-12863108.html (Accessed July 12, 2022).

[10] Mo S Shi J. The Psychological Consequences of the COVID-19 on Residents and Staff in Nursing Homes. Work Aging Retire (2020) 6(4):254–9. 10.1093/workar/waaa021 34192005 PMC7665707

[11] Simonetti A Pais C Jones M Cipriani MC Janiri D Monti L Neuropsychiatric Symptoms in Elderly with Dementia during COVID-19 Pandemic: Definition, Treatment, and Future Directions. Front Psychiatry (2020) 11(579842):579842. 10.3389/fpsyt.2020.579842 33132939 PMC7550649

[12] Pérez-Rodríguez P Díaz de Bustamante M Aparicio Mollá S Arenas MC Jiménez-Armero S Lacosta Esclapez P Functional, Cognitive, and Nutritional Decline in 435 Elderly Nursing Home Residents after the First Wave of the COVID-19 Pandemic. Eur Geriatr Med (2021) 12:1137–45. 10.1007/s41999-021-00524-1 34165775 PMC8222945

[13] Wallace CL Wladkowski SP Gibson A White P. Grief during the COVID-19 Pandemic: Considerations for Palliative Care Providers. J Pain Symptom Manage (2020) 60(1):e70–6. 10.1016/j.jpainsymman.2020.04.012 PMC7153515 32298748

[14] Colorado Department of Public Healt & Environment. Residential Care Facilities - Outdoor Visitation | Colorado COVID-19 Updates (2024). Available from: https://covid19.colorado.gov/outdoor-visitation (Accessed July 12, 2022).

[15] Nursing Home Reopening Recommendations for State and Local Officials. CMS (2022) Available from: https://www.cms.gov/medicareprovider-enrollment-and-certificationsurveycertificationgeninfopolicy-and-memos-states-and/nursing-home-reopening-recommendations-state-and-local-officials (Accessed July 12, 2022).

[16] Radiotelevisione Svizzera Italiana (RSI). Un Parlatorio Per Gli Anziani (2020). Available from: https://www.rsi.ch/news/ticino-e-grigioni-e-insubria/Un-parlatorio-per-gli-anziani-12866687.html (Accessed July 28, 2021).

[17] Corriere del Ticino. Anziani Meno Soli Grazie Alle Videochiamate (2020). Available from: https://www.cdt.ch/ticino/bellinzona/anziani-meno-soli-grazie-alle-videochiamate-FK2480351 (Accessed July 28, 2021).

[18] Radiotelevisione Svizzera Italiana (RSI). Tornano Gli Abbracci (2020). Available from: https://www.rsi.ch/news/ticino-e-grigioni-e-insubria/Tornano-gli-abbracci-13117383.html (Accessed July 12, 2022).

[19] Ufficio federale della sanità pubblica (UFSP). Allentamenti e Inasprimenti dei Provvedimenti Nazionali Stato Delle Tabelle: Dal 27 Aprile al 2 Novembre 2020 (2020). Available from: https://www.newsd.admin.ch/newsd/message/attachments/61507.pdf (Accessed July 18, 2021).

[20] Monin JK Ali T Syed S Piechota A Lepore M Mourgues C Family Communication in Long-Term Care During a Pandemic: Lessons for Enhancing Emotional Experiences. Am J Geriatr Psychiatry (2020) 28(12):1299–307. 10.1016/j.jagp.2020.09.008 33004262 PMC7486818

[21] Verbeek H Gerritsen DL Backhaus R de Boer BS Koopmans RTCM Hamers JPH. Allowing Visitors Back in the Nursing Home During the COVID-19 Crisis: A Dutch National Study into First Experiences and Impact on Well-Being. J Am Med Dir Assoc (2020) 21(7):900–4. 10.1016/j.jamda.2020.06.020 32674816 PMC7294280

[22] Chu CH Yee A Stamatopoulos V. Poor and Lost Connections: Essential Family Caregivers’ Experiences Using Technology With Family Living in Long-Term Care Homes during COVID-19. J Appl Gerontol (2022) 41(6):1547–56. 10.1177/07334648221081850 35416076 PMC9014337

[23] Zhao X Zhang D Wu M Yang Y Xie H Li Y Loneliness and Depression Symptoms Among the Elderly in Nursing Homes: A Moderated Mediation Model of Resilience and Social Support. Psychiatry Res (2018) 268:143–51. 10.1016/j.psychres.2018.07.011 30025285

[24] Zhang D Cheung G Cullum S Ng L. Navigating WeChat in COVID Times as a Chinese Care Home Resident. J Long-term Care (2022) 234–43. 10.31389/jltc.153

[25] Lindgren CL Murphy AM. Nurses’ and Family Members’ Perceptions of Nursing Home Residents’ Needs. J Gerontol Nurs (2021) 28(8):45–53. 10.3928/0098-9134-20020801-10 12219553

[26] Hockley J Hafford-Letchfield T Noone S Mason B Jamieson L Iversholt R COVID, Communication and Care Homes: A Staffs’ Perspective of Supporting the Emotional Needs of Families. J Long-term Care (2021) 167–76. 10.31389/jltc.74

[27] Veiga-Seijo R Miranda-Duro M del C Veiga-Seijo S. Strategies and Actions to Enable Meaningful Family Connections in Nursing Homes During the COVID-19: A Scoping Review. Clin Gerontol (2022) 45(1):20–30. 10.1080/07317115.2021.1937424 34170785

[28] Rutten JER Backhaus R Ph Hamers J Verbeek H. Working in a Dutch Nursing Home during the COVID-19 Pandemic: Experiences and Lessons Learned. Nurs Open (2022) 9(6):2710–9. 10.1002/nop2.970 34227749 PMC8441710

[29] Havaei F MacPhee M Keselman D Staempfli S. Leading a Long-Term Care Facility through the COVID-19 Crisis: Successes, Barriers and Lessons Learned. Healthc Q (2021) 23(4):28–34. 10.12927/hcq.2020.26396 33475489

[30] Badawy A Solberg M Obstfelder AU Alnes RE. Improvised Use of a Digital Tool for Social Interaction in a Norwegian Care Facility during the COVID-19 Pandemic: An Exploratory Study. BMC Health Serv Res (2022) 22(136):136. 10.1186/s12913-022-07526-0 35105344 PMC8804078

[31] White EM Wetle TF Reddy A Baier RR. Front-Line Nursing Home Staff Experiences During the COVID-19 Pandemic. J Am Med Dir Assoc (2021) 22(1):199–203. 10.1016/j.jamda.2020.11.022 33321076 PMC7685055

[32] Juul A Wilding R Baldassar L. The Best Day of the Week: New Technology Enhancing Quality of Life in a Care Home. Int J Environ Res Public Health (2019) 16(6):1000. 10.3390/ijerph16061000 30893945 PMC6466428

[33] Bollig G Gjengedal E Rosland JH. Nothing to Complain About? Residents’ and Relatives’ Views on a “Good Life” and Ethical Challenges in Nursing Homes. Nurs Ethics (2016) 23(2):142–53. 10.1177/0969733014557719 25488765 PMC4786778

[34] Anderson DC Grey T Kennelly S O’Neill D. Nursing Home Design and COVID-19: Balancing Infection Control, Quality of Life, and Resilience. J Am Med Dir Assoc (2020) 21(11):1519–24. 10.1016/j.jamda.2020.09.005 33138934 PMC7603995

[35] Bollig G Schmidt G Rosland JH Heller A. Ethical Challenges in Nursing Homes – Staff’s Opinions and Experiences With Systematic Ethics Meetings With Participation of Residents’ Relatives. Scand J Caring (2015) 29(4):810–23. 10.1111/scs.12213 25918868

[36] Preshaw DH Brazil K McLaughlin D Frolic A. Ethical Issues Experienced by Healthcare Workers in Nursing Homes: Literature Review. Nurs Ethics (2016) 23(5):490–506. 10.1177/0969733015576357 25870176

[37] Heggestad AKT Nortvedt P Slettebø Å. Dignity and Care for People With Dementia Living in Nursing Homes. Dement Lond Engl (2015) 14(6):825–41. 10.1177/1471301213512840 24381212

[38] Ryan AA Scullion HF. Family and Staff Perceptions of the Role of Families in Nursing Homes. J Adv Nurs (2000) 32(3):626–34. 10.1046/j.1365-2648.2000.01521.x 11012805

[39] Lao SSW Low LPL Wong KKY. Older Residents’ Perceptions of Family Involvement in Residential Care. Int J Qual Stud Health Well-being (2019) 14(1):1611298. 10.1080/17482631.2019.1611298 31072244 PMC6522931

