# Supplementary material for: Navigating Communication in Nursing Homes During COVID-19: Perspectives From Families, Healthcare Professionals, and Managers in Southern Switzerland—A Qualitative Study
Source: Int J Public Health. 2024 Oct 22;69:1606583. doi: 10.3389/ijph.2024.1606583 (PMC11535700; doi:10.3389/ijph.2024.1606583)
Supplement: Supplementary file 1 [file DataSheet1.pdf]

International journal of public health

Communicating with and about nursing homes' residents during the first wave of COVID-19 pandemic. A qualitative study in southern Switzerland

## Griglia di intervista

Data:
Luogo (Casa Anziani):
Intervistatore/rice:
Codice intervistato:
Genere:
Gruppo (familiare, infermiere, medico, direttore):
*Se curante*
Ruolo:
Anni di esperienza:

**Introduzione**

Buongiorno e grazie per aver accettato di partecipare a questa intervista. Facciamo parte della XX e stiamo conducendo uno studio in collaborazione con XX che ha l'obbiettivo di esplorare i vissuti di curanti e familiari dei residenti in Casa per Anziani durante la chiusura dovuta all'emergenza sanitaria da COVID-19. In particolare siamo interessati a comprendere quali misure messe in atto dalle Case per Anziani hanno facilitato o ostacolato la soddisfazione dei familiari durante l'isolamento.

I risultati della ricerca permetteranno di fornire informazioni utili alle case per anziani rispetto all'efficacia delle misure messe in atto e da applicare per un eventuale secondo picco di pandemia. I risultati saranno pubblicati in riviste scientifiche ma non sarà in alcun modo possibile risalire alla sua identità.

L'intervista durerà circa 30 minuti, la sua partecipazione è volontaria e può decidere di interromperla in qualsiasi momento senza fornire giustificazioni. Se lei è d'accordo, registriamo l'intervista così da poter analizzare successivamente quello che ci siamo detti. Le garantiamo che tutte le informazioni saranno trattate nel pieno rispetto della privacy e con la massima confidenzialità. Soltanto il team di ricerca avrà accesso alle sue informazioni.

Prima di entrare nel vivo dell'intervista le consegnerò un formulario per il consenso. Le lascerò una copia sia del formulario del consenso informato che dell'informativa. Poi partiremo da alcune domande generali verso altre più specifiche e toccheremo vari aspetti quali la comunicazione della chiusura delle case per anziani, il suo parere rispetto alla chiusura, il suo vissuto e i rapporti tra casa anziani e familiari.

Ci sono domande a parte sua?

International journal of public health

Communicating with and about nursing homes' residents during the first wave of COVID-19 pandemic. A qualitative study in southern Switzerland

**Domande**

1. Come ha appreso la notizia della chiusura delle case per anziani?
    a. Da chi ha appreso la notizia? (media, direttore CpA, altro)
    b. Come gli è stato comunicato?
    c. Qual è stata la sua reazione alla notizia della chiusura delle Case per Anziani?
    d. (SOLO per i direttori e operatori): Com'è stata comunicata la notizia della chiusura ai familiari?

2. Che cosa ne pensa della chiusura delle case per anziani?
    a. La chiusura delle CpA le è sembrata proporzionata?
    b. Si aspettava una tale decisione?
    c. Qual era la sua percezione della situazione sanitaria prima della chiusura delle CpA?

3. Come ha vissuto la chiusura delle Casa per Anziani?
    a. C'è qualcosa che ha influito sul suo vissuto?
    b. Ci sono stati accorgimenti, informazioni, azioni da parte della casa per anziani che hanno influito sul suo vissuto?
    c. (SOLO per i direttori e operatori): Secondo lei e secondo la sua esperienza com'è stata vissuta la chiusura dalla parte dei familiari?

4. Come sono stati i rapporti fra casa anziani e familiari durante l'isolamento?
    a. (SOLO Direttori e operatori) Com'è stata la comunicazione con il familiare?
    b. (SOLO familiare): Com'è stata la comunicazione con i curanti e la casa per anziani?
    c. Quali misure adottate nella comunicazione fra residente-familiare familiare-casa per anziani sono state messe in atto?
    d. Che cosa ne pensa di queste misure? Sono state efficaci?
    e. Si aspettava altro dalle Case per Anziani? Cos'altro avrebbe potuto fare la Casa per Anziani?

5. C'è qualcos'altro che vuole raccontarci?

La ringraziamo per la sua partecipazione. Per qualsiasi domanda e precisazione può contattarci via email XX o al numero telefonico XX
